# Correlates of Delayed Diagnosis among Human Immunodeficiency Virus-Infected Pulmonary Tuberculosis Suspects in a Rural HIV Clinic, South Africa

**DOI:** 10.1155/2012/827148

**Published:** 2012-06-13

**Authors:** Respicious Boniface, Mosa Moshabela, Rose Zulliger, Peter MacPherson, Peter Nyasulu

**Affiliations:** ^1^Epidemiology and Biostatistics Division, School of Public Health, Faculty of Health Sciences, University of The Witwatersrand, 7 York Road, Parktown, Johannesburg 2193, South Africa; ^2^Centre for Infectious Disease Epidemiology and Research (CIDER), University of Cape Town, Cape Town 7925, South Africa; ^3^Liverpool School of Tropical Medicine, Liverpool L35 QA, UK; ^4^Malawi-Liverpool-Wellcome Trust Clinical Research Programme, Blantyre, Malawi

## Abstract

*Background. *Delay in pulmonary tuberculosis (PTB) diagnosis is one of the major factors that affect outcome and threatens continued spread of tuberculosis. This study aimed at determining factors associated with delayed PTB diagnosis among human immunodeficiency virus (HIV) infected individuals. *Methods. *A retrospective observational study was done using clinic records of HIV-infected PTB suspects attending an HIV/AIDS clinic at Tintswalo rural hospital in South Africa (SA) between January 2006 and December 2007. Using routine clinic registers, 480 records were identified. *Results. *PTB diagnosis delay was found among 77/176 (43.8%) of the patients diagnosed with PTB. The mean delay of PTB diagnosis was 170.6 days; diagnosis delay ranged 1–30 days in 27 (35.1%) patients, 31–180 days in 24 (33.8%) patients; 24 (31.2%) patients remained undiagnosed for ≥180 days. Independent factors associated with delayed diagnosis were: older age >40 years (Odds Ratio (OR) 3.43, 95% CI 1.45–8.08) and virological failure (OR 2.72, 95% CI 1.09–6.74). *Conclusion. *There is a considerable delayed PTB diagnosis among HIV-infected patients in rural SA. Older patients as well as patients with high viral load are at a higher risk of PTB diagnosis delay. Therefore efforts to reduce PTB diagnosis delay need to emphasised.

## 1. Background

An estimated one-third of the world's population is infected with tuberculosis (TB) [1]. The human immunodeficiency virus (HIV) pandemic has resulted in dramatic increases in TB case notification rates, particularly in resource-limited settings [2]. This is because people living with HIV have a much greater risk of developing active TB than HIV-uninfected individuals [3]. South Africa is one of the countries most heavily affected by the dual HIV and TB epidemics [4], with an estimated 31% of all global TB cases occurring among HIV-positive individuals [5]. For example, in Gugulethu, a township in Cape Town, over half of antiretroviral therapy (ART) clinic attendees had previously been treated for TB, one-quarter were diagnosed with active TB, and a further 10% developed TB during the first year following initiation onto lifelong ART [6].

In resource-limited settings, where the majority of HIV-TB cases occur, TB diagnostics are frequently limited to sputum smear microscopy, despite the increased likelihood of smear negative disease among immunosuppressed patients [7]. This scenario complicates TB diagnosis and frequently results in delay in TB diagnosis in HIV-infected patients [8]. This delayed detection often leads to increased mortality and treatment complications for patients [9]. In addition, undiagnosed TB in patients starting ART may result in symptomatic immune reconstitution inflammatory syndrome during early ART phase because of unmasking of TB disease by ART [10]. We have previously reported on high mortality due to TB among ART initiators in this community [11]. Reducing delays in PTB diagnosis could improve patients' health outcomes and minimise public risk of exposure to TB in the community. The purpose of this study was to investigate factors associated with the delay in TB diagnosis among HIV patients following entry into public sector HIV services in rural South Africa.

## 2. Methods

### 2.1. Study Setting

This study was conducted at Rixile Clinic, a dedicated nongovernmental-organisation-(NGO-) supported, rural HIV clinic situated at Tintswalo hospital in Bushbuckridge, Mpumalanga, South Africa. Three HIV-trained doctors and six primary health care nurses provide comprehensive care for HIV patients at the clinic, including provision of ART, and screening for and management of TB, opportunistic infections, and sexually transmitted infections. The clinic was accredited for provision of ART in October 2005. Since then more than 2,000 individuals have been initiated on ART. Bushbuckridge is a densely populated rural area with a population of approximately 600,000 people living in 133 villages. The region is one of 13 rural nodal areas of extreme poverty in South Africa.

### 2.2. Study Design

 This was a retrospective cohort study of PTB suspects at Rixile Clinic. Data were extracted from the ART clinic records of patients identified as PTB suspects from the PTB suspects register over a two-year period from January 2006 to December 2007. 

### 2.3. Study Population

 The study population was comprised of adults (18 years and older) at the time of identification as a PTB suspect and with confirmed HIV infection. All patients had attended the Rixile clinic at least once. PTB suspects were defined as patients who were provided with sputum jars for Acid-Fast Bacilli screening for PTB. 

### 2.4. Data Collection and Management

 Trained research assistants extracted data from the PTB suspects register and from patients' ART clinic medical records using a structured data collection tool. Data were abstracted on the following variables: sociodemographic factors, presenting symptoms, timing, and results of TB investigations, diagnosis category, date and regimen of TB treatment initiated, and details of HIV treatment, including ART initiation. Data were double-entered into Epi info version 3.5.1 (CDC, Atlanta, USA). Statistical analyses were conducted using STATA version 11 (Statacorp, College Station, USA). 

### 2.5. Definition of TB Diagnosis Delay

TB diagnostic delay was defined as any diagnosis taking longer than 56 days from the time of requesting the first sputum examination. Although this definition is unconventional, it allows for the time required to complete all diagnostic examinations, including sputum culture, which is routinely requested among HIV-positive TB suspects with smear-negative results attending services at Rixile HIV clinic [12].

### 2.6. Statistical Analysis

 Frequency distributions were used to describe categorical variables, and medians and interquartile ranges were used for continuous variables. The dependent variable diagnosis delay was categorized as a dichotomous variable (delay or nodelay). Bivariate associations were described using Chi-square tests. Variables that demonstrated significant association (*P* ≤ 0.1) with TB diagnosis delay were entered into a multivariate Cox proportional hazards regression model. 

### 2.7. Ethical Consideration

 Ethical approval for the study was obtained from the University of Witwatersrand Human Research Ethics Committee. Individual informed consent was not undertaken as the study used routinely collected clinic data.

## 3. Results

**Figure 1:**
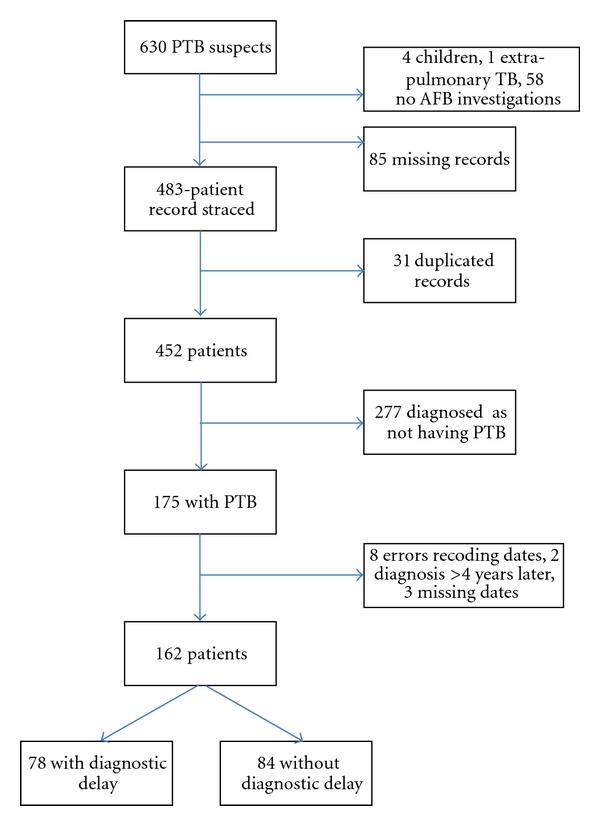
Cohort Flowchart **4 younger than 18 years, 1 with extrapulmonary TB, 58 with no AFB investigation requested. ***31 observations were duplicates ****8 with negative time interval to TB diagnosis, 2 with time interval to TB diagnosis >4 years, 3 with missing TB diagnosis date.

In the two-year study period, 630 individuals were identified as PTB suspects and recorded in the PTB suspect register. Sixty-two were excluded for the following reasons: 4 were younger than 18 years old, 42 did not return their sputum jars and did not attend any subsequent review, 1 had extra pulmonary TB, and there was no information on the outcome of TB diagnosis for 15 patients. Eighty-five patients' medical records were missing and 31 were duplicates. Thus, a total of 452 PTB suspects were included in the study.  Figure 1 summarizes the patients' inclusion cascade.

The demographic information of the 452 patients included in the preliminary analysis is summarized in Table 1. The ages of the patients ranged from 23 to 96 years old with a median age of 41 years (interquartile range (IQR) = 35–49). Seventy percent (316/452) of the participants were women; 175/446 (39.2%) were not married, 191/447 (42.7%) had attended primary school only, and more than half 403/449 (89.7%) were unemployed. 

### 3.1. PTB Cases Diagnosed

Of the 452 PTB suspects, 162 (35.8%) PTB cases were diagnosed. Of those diagnosed with PTB, 40 (24.7%) were diagnosed on the basis of sputum smear positivity, 84 (51.9%) through positive sputum culture, and 38 (23.5%) through chest radiograph.

### 3.2. Outcomes of PTB Treatment

Out of the 162 patients diagnosed with PTB and started on TB treatment, 86 (53.1%) completed treatment, 14 (8.6%) died, 34 (21.0%) were lost to follow up, and 28 (17.3%) were still on treatment at time of data extraction. 

### 3.3. Delay in Diagnosis of PTB

The median delay from presentation with PTB symptoms to PTB diagnosis was 55 days (IQR = 20–302). Delay in diagnosis of PTB of more than 56 days was observed among 78 (48.2%) of the 162 patients diagnosed with PTB. The overall delay was 57 to 86 days in 27 (34.6%), 87 to 223 days in 26 (33.3%), and >236 days in 25 (32.1%) of the patients diagnosed with PTB. The diagnosis was made by clinical history plus chest X-ray for 19 of the 78 (24.7%) and by sputum smear microscopy for 12 (15.4%) The majority of patients with diagnostic delay had their diagnosis made by sputum culture 47/78 (60.3%) (Table 2).

### 3.4. Factors Associated with TB Diagnosis Delay

Univariate analysis showed patient's age (*P* = 0.006), method of TB diagnosis (*P* = 0.025), detectable HIV viral load (*P* = 0.025), and ART use at the time of PTB diagnosis (*P* ≤ 0.001), to be significantly associated with TB diagnosis delay (Table 3). Age, viral load and ART use at the time of PTB diagnosis remained statistically significant in the multivariate analysis. Patients who were older than 40 years had a 1.57 times greater risk of TB diagnostic delay than those aged 18–40 years. The risk of TB diagnostic delay was 1.89 times greater in those with HIV viral load ≥400 copies/mL than patients with HIV viral load ≤400 copies/mL; however the observed association was not statistically significant *P* = 0.06. Those who were on ART at the time of PTB diagnosis were 51% less likely to have experienced diagnostic delay than those who were not on ART (Table 4).

## 4. Discussion

A large proportion of PTB patients experienced diagnostic delays of greater than 56 days. These findings have important implications for informing current Stop-TB efforts aimed at reducing global TB incidence and mortality. TB diagnosis delay was associated with viral load ≥400 copies/mL. Delayed detection leads to patients presenting with advanced TB which promotes HIV/AIDS disease progression by accelerating viral multiplication [13].

TB diagnostic delay was significantly associated with patient's age. Older patients (>40 years) were at a much higher risk of TB diagnostic delay compared with those younger than 40 years. This is likely to be due to coexisting medical conditions in the elderly persons such as heart diseases and chronic chest problems, all of which may contribute to the difficulty of diagnosing TB and can thus result in delayed diagnosis and treatment [14, 15]. 

The majority of patients with diagnostic delay in this study (60.3%) were diagnosed with PTB by sputum culture. This may be because the study was conducted among HIV-infected patients, and studies have reported reduced diagnostic sensitivity of sputum smears and chest radiography in HIV infected patients [7]. Given the high percentage of patients diagnosed by TB culture, these findings support the practice of regular sputum culture tests to confirm sputum negative smears in ART clinics. Those who were not on ART at the time of PTB diagnosis were more likely to have delayed diagnosis than those who were on ART. ART unmasks TB in HIV-infected patients with subclinical disease making it easier to detect and diagnose [10]. 

There are several factors that have been reported from previous studies that influences delay in TB diagnosis. These could be patient, clinician, or health system related. Patient-related factors include choice of health care provider, stigma, alcoholism, substance abuse, general state of poor health, tobacco smoking, and chronic cough with blood-stained sputum [16–26]. Clinician-related factors include: poor training of clinical personnel in TB diagnosis, low level of TB awareness among private practitioners, and lack of knowledge of TB diagnosis among traditional healers [20, 22, 24–29]. Health system factors that play a role in delayed TB diagnosis include lack of diagnostic facilities and low access to health care services [26, 30].

High viral load is a clinical indicator of declining immunological function of an HIV-infected individual which makes them to be at higher risk of developing active TB. In this era of ongoing HIV epidemic, diagnosis and clinical management of active TB poses a major challenge in patients with dual infection [31]. Due to immunosuppression, there is lack of pulmonary cavitations resulting in low bacillary concentration in sputum giving rise to high rate of negative sputum smear for AFB as well as negative chest radiographic findings. This has led to an over reliance of TB diagnosis through sputum culture which is an expensive and slow diagnostic method [32, 33]. This could be the reason why our study showed that a positive relationship exists between diagnostic delay and decline in immune function (viral load >400 copies/mL).

In considering the findings of this study it is important to bear in mind the following limitations: firstly, as this cohort was recruited from an ART clinic, they may have some differences in characteristics to those participants in hospitals, public health clinics, and the general community. Secondly, the study population did not include PTB suspects who were younger than 18 years, and 85 patients were excluded due to missing medical records. It is possible that some in both groups had active TB. Thirdly, accuracy of interpretation of routinely offered TB diagnostic tests (sputum smear and chest radiographs) depends on the skills and experience of the attending clinician. Interpretation of these tests is often subjective and prone to error; hence some of the results might have been incorrect. Fourthly, some patients in this cohort 42/630 (6.7%) did not return their sputum jars (lost to follow-up) and, therefore, were not investigated for AFB. It is possible that some of these patients had TB or had died of TB. Lastly we did not have data documenting the duration of TB symptoms prior to presenting at a health facility, therefore we would not know length of diagnosis delay attributed to TB patient's health seeking behaviour. The exclusion of patients who were lost-to-follow-up and the definition of delay from presentation at health system, rather than start of symptoms, make these findings conservative as the true proportion of patients experiencing diagnosis delays would be higher than what has been reported in this study.

## 5. Conclusion

The diagnostic delays for PTB demonstrated in this study, as well as the difficulty of diagnosing TB in HIV-infected patients, point to the need for improved screening of TB suspects in ART clinics. Novel TB diagnostic methods such as Gene-Xpert, which are rapid, highly sensitive, and specific, should be promoted in primary health care facilities in order to ensure the timely diagnosis of PTB. 

## Figures and Tables

**Table 1:** Socioeconomic characteristics of study participants.

Characteristic	N	(%)
Marital status		
Divorced	81	18.2
Married	131	29.4
Never married	175	39.2
Widowed	59	13.2
Total	446	100.0
Education level		
No education	90	20.1
Primary	191	42.8
Secondary	152	34.0
Tertiary	14	3.1
Total	447	100.0
Occupation status		
Salaried worker	46	10.3
Unemployed and willing to work	142	31.6
Unemployed and not willing to work	261	58.1
Total	449	100.0
Cigarette smoker		
No	359	80.7
Yes	86	19.3
Total	445	100.0
Alcohol drinker		
No	355	79.9
Yes	89	20.1
Total	444	100.0
Main material walls of house		
Block cement	229	51.0
Brick	115	25.6
Mud	105	23.4
Total	449	100.0
People in households		
1 to 5	239	53.1
6 to 10	187	41.6
>10	24	5.3
Total	450	100.0

N: number of study participants in each variable.

**Table 2:** Diagnosis delay duration by method of diagnosis.

Method of TB diagnosis	57 to 86 days delay ^∗∗^N (%)	87 to 236 days delay ^∗∗^N (%)	>236 days delay ^∗∗^N (%)	Total Number (%)	*P* value
Clinical history and chest X-ray	4 (15.3)	5 (19.2)	10 (40)	19 (24.7)	0.016
Sputum smear positive	3 (11.5)	2 (7.7)	7 (28)	12 (15.6)	
Sputum culture	20 (74.1)	19 (73.1)	8 (32)	47 (60.3)	
Total	27 (100)	26 (100)	25 (100)	78 (100)	

^∗∗^N: number of TB patients diagnosed within each time duration.

**Table 3:** Comparison of sociodemographic and clinical factors of participants with TB diagnosis delay.

Characteristic	Diagnosis delay number (%)	No delay number (%)	*P* value
Patient age			
18 to 40 years	33 (42.9)	54 (64.3)	0.006^∗∗^
>40 years	44 (57.1)	30 (35.7)	
Sex			
Female	49 (62.9)	58 (69.0)	0.403
Male	29 (37.1)	26 (31.0)	
Marital status			
Divorced	13 (16.9)	13 (15.4)	0.592
Married	25 (32.5)	21 (25.0)	
Never married	27 (35.1)	38 (45.2)	
Widowed	12 (15.5)	12 (14.3)	
Occupation status			
Salaried worker	9 (11.7)	7 (8.3)	0.538
Unemployed and willing to work	21 (27.3)	29 (34.5)	
Unemployed and not willing to work	47 (61.0)	48 (57.2)	
Smoke cigarette			
No	57 (74.0)	66 (78.6)	0.410
Yes	20 (26.0)	18 (21.4)	
Alcohol drinking			
No	57 (74.0)	65 (79.3)	0.434
Yes	20 (26.0)	17 (20.7)	
Education level			
No education	16 (20.8)	14 (16.7)	0.320
Primary	37 (48.0)	33 (39.2)	
Secondary	23 (29.9)	33 (39.3)	
Tertiary	1 (1.3)	4 (4.8)	
Main material walls of house			
Block cement	38 (49.3)	47 (56.0)	0.377
Brick	19 (24.7)	22 (26.1)	
Mud	20 (26.0)	15 (17.9)	
Method of TB diagnosis			
Clinical history and CXR	19 (24.7)	19 (22.9)	0.025^∗∗^
Sputum AFB	12 (15.6)	28 (33.7)	
Sputum culture	46 (59.7)	36 (43.4)	
WHO-HIV clinical stage			
1	2 (3.6)	2 (2.9)	1.000
2	9 (16.4)	16 (23.5)	
3	42 (76.4)	44 (64.8)	
4	2 (3.6)	6 (8.8)	
CD4 count (cells/mm^3^)			
<50	4 (6.9)	1 (1.8)	0.193
50–200	15 (25.9)	10 (17.9)	
>200	39 (67.2)	45 (80.3)	
Viral load (copies/mL)			
≤400	31 (60.8)	39 (81.3)	0.025^∗∗^
>400	20 (39.2)	9 (18.7)	
BMI (Kg/m^2^)			
Underweight	16 (25.4)	15 (22.4)	0.828
Normal weight	34 (53.9)	38 (56.7)	
Overweight	12 (19.0)	11 (16.4)	
Obese	1 (1.5)	3 (4.5)	
ART use at PTB diagnosis			
No	38 (50.0)	62 (77.5)	≤0.001^∗∗^
Yes	38 (50.0)	18 (22.5)	
TB treatment outcome			
Completed treatment	41 (52.5)	45 (53.6)	0.640
Died	7 (9.1)	7 (8.3)	
Lost to follow up	14 (17.9)	20 (23.8)	
Still on treatment	16 (20.5)	12 (14.3)	

^∗∗^ *P* value ≤ 0.05.

**Table 4:** Univariate and multivariate Cox proportional hazards regression analysis.

Characteristic	Univariate HR (95% CI)	*P*-value	Multivariate HR (95% CI)	*P* value
Patient age				
18 to 40 years	1		1	
>40 years	1.8 (0.53–1.32)	0.05	1.57 (0.25–0.90)	0.02
Sex				
Female	1			
Male	1.26 (0.79–2.03)	0.33	—	—
Education level				
No education	1			
Primary	2.62 (1.28–5.34)	0.25		
Secondary	7.09 (0.87–7.56)	0.17		
Tertiary	1.54 (0.79–2.95)	0.19		
Method of TB diagnosis				
History and CXR	1			
Smear positive	0.83 (0.48–2.14)	0.95	—	—
Sputum culture positive	2.09 (1.19–3.65)	0.09		
Viral load (copies/mL)				
<400	1		1	
>400	2.3 (0.87–1.56)	0.07	1.89 (0.74–1.63)	0.06
CD4 Count (cells/mm^3^)				
<50	1			
50–200	0.56 (0.18–1.72)	0.31	—	—
>200	0.64 (0.20–1.64)	0.42		
ART use at PTB diagnosis				
No	1		1	
Yes	0.82 (1.05–1.3)	0.04	0.49 (0.36–0.97)	0.04
TB treatment outcome				
Completed treatment	1			
Died	2.42 (1.07–5.47)	0.03	—	—
Lost to follow-up	0.92 (0.49–1.73)	0.81		
Still on treatment	0.79 (0.43–1.46)	0.45		

HR: hazard ratio; CI: confidence interval.
